# The Gustave Roussy Immune (GRIm)-Score Variation Is an Early-on-Treatment Biomarker of Outcome in Advanced Non-Small Cell Lung Cancer (NSCLC) Patients Treated with First-Line Pembrolizumab

**DOI:** 10.3390/jcm10051005

**Published:** 2021-03-02

**Authors:** Edoardo Lenci, Luca Cantini, Federica Pecci, Valeria Cognigni, Veronica Agostinelli, Giulia Mentrasti, Alessio Lupi, Nicoletta Ranallo, Francesco Paoloni, Silvia Rinaldi, Linda Nicolardi, Andrea Caglio, Sophie Aerts, Alessio Cortellini, Corrado Ficorella, Rita Chiari, Massimo Di Maio, Anne-Marie C. Dingemans, Joachim G. J. V. Aerts, Rossana Berardi

**Affiliations:** 1 Department of Medical Oncology, Università Politecnica delle Marche, AOU Ospedali Riuniti Ancona, 60126 Ancona, Italy; edoardo.lenci@hotmail.it (E.L.); lucacantini.med@gmail.com (L.C.); peccifede91@gmail.com (F.P.); valeriacognigni93@gmail.com (V.C.); veroagostinelli@gmail.com (V.A.); giulia.mentrasti17@gmail.com (G.M.); lupialessio2@gmail.com (A.L.); nicolettaran@gmail.com (N.R.); fapaoloni@gmail.com (F.P.); sylvya.rinaldi85@gmail.com (S.R.); 2 Department of Pulmonary Medicine, Erasmus MC Rotterdam, 3015 GD Rotterdam, The Netherlands; aertsmsophie@gmail.com (S.A.); a.dingemans@erasmusmc.nl (A.-M.C.D.); j.aerts@erasmusmc.nl (J.G.J.V.A.); 3 Erasmus MC Cancer Institute, Erasmus MC Rotterdam, 3015 GD Rotterdam, The Netherlands; 4 Medical Oncology, Ospedali Riuniti Padova Sud, 35043 Monselice, Italy; linda.nicolardi@aulss6.veneto.it (L.N.); rita.chiari@aulss6.veneto.it (R.C.); 5 Department of Oncology, University of Turin, Ordine Mauriziano Hospital, 10128 Torino, Italy; andrea.caglio@edu.unito.it (A.C.); massimo.dimaio@unito.it (M.D.M.); 6 Medical Oncology, St Salvatore Hospital, 67100 L’Aquila, Italy; alessiocortellini@gmail.com (A.C.); corrado.ficorella@univaq.it (C.F.); 7 Department of Biotechnology and Applied Clinical Sciences, University of L’Aquila, 67100 L’Aquila, Italy

**Keywords:** GRIm-Score, immunotherapy, first-line, NSCLC, pembrolizumab

## Abstract

Background: The Gustave Roussy Immune (GRIm)-Score takes into account neutrophil-to-lymphocyte ratio (NLR), serum albumin concentration and lactate dehydrogenase (LDH) and its prognostic value has been investigated in patients treated with immune check-point inhibitors (ICIs). To further assess the prognostic and predictive value of baseline GRIm-Score (GRImT0) in advanced non-small cell lung cancer (aNSCLC) patients, we separately investigated two cohorts of patients treated with first-line pembrolizumab or chemotherapy. We also investigated whether GRIm-Score at 45 days since treatment initiation (GRImT1) and GRIm-Score difference between the two timepoints may better predict clinical outcomes (GRImΔ = GRImT0 − GRImT1). Methods: We retrospectively evaluated 222 aNSCLC patients: 135 treated with pembrolizumab and 87 treated with chemotherapy as the first-line regimen. NLR, serum albumin and LDH concentrations were assessed at T0 and at T1. According to the GRIm-Score, patients were assigned 1 point if they had NLR > 6, LDH > upper limit normal or albumin < 3.5 g/dL. Patients with a GRIm-Score < 2 were considered as having a low Score. Results: In both cohorts, no difference in terms of overall survival (OS) between patients with low and high GRImT0 was found. Otherwise, median OS and progression free survival (PFS) of the low GRImT1 group were significantly longer than those of the high GRImT1 group in pembrolizumab-treated patients, but not in the CHT cohort (pembrolizumab cohort: low vs. high; median OS not reached vs. 9.2 months, *p* = 0.004; median PFS 10.8 vs. 2.3 months, *p* = 0.002). Patients receiving pembrolizumab with stable/positive GRImΔ had better OS (median OS not reached vs. 12.0 months, *p* < 0.001), PFS (median PFS 20.6 vs. 2.6 months, *p* < 0.001) and objective response rate (58.2% vs. 7.6%, *p* = 0.003) compared to patients with negative GRImΔ. Conclusion: Our data shown that GRImT1 and GRImΔ are more reliable peripheral blood biomarkers of outcome compared to GRImT0 in aNSCLC patients treated with pembrolizumab and might represent useful biomarkers to drive clinical decisions in this setting.

## 1. Introduction

Until 2015, chemotherapy represented the only available systemic treatment for patients affected by advanced non-oncogene addicted non-small cell lung cancer (aNSCLC). Platinum-based doublet chemotherapy was the first-line standard approved regimen, with an estimated median overall survival of 11–16 months [1,2,3].

Recently, immune check-point inhibitors (ICIs) have remarkably changed this scenario, leading to a significant benefit in patient clinical outcome with respect to chemotherapy [4,5,6,7]. Indeed, the use of pembrolizumab in first-line treatment of aNSCLC patients with a programmed death ligand 1 (PD-L1) tumor proportion score of 50% or greater led to an estimated median overall survival (OS) of 30 months within clinical trials [8].

Despite the well-known survival benefit and the manageable toxicity profile [9], pembrolizumab does not represent the right choice for every patient. A proportion of patients between 4% and 29% experience hyperprogression under immunotherapy [10] and the identification of prognostic and predictive clinical parameters in this setting could help clinical management [11].

Routine baseline blood parameters including upper limit normal (ULN) LDH, low albumin levels and others such as high neutrophil to lymphocyte ratio (NLR), have already been associated with a worse outcome in different types of cancer patients, including aNSCLC patients treated with ICIs or chemotherapy [12,13,14,15]. Other blood-derived scores, such as the Lung Immune Prognostic Index (LIPI) [16] or the Royal Marsden Hospital prognostic score [17], also showed an association with survival outcomes in aNSCLC patients treated with ICIs. Similarly, blood parameters variations might represent interesting tools for driving treatment decisions [18,19].

The Gustave Roussy Immune (GRIm)-Score was initially validated on patients enrolled into phase 1 trials with ICIs [20]. GRIm-Score is a combination of NLR, albumin serum level and LDH that stratifies patients in classes of high and low risk (patients with GRIm-Score > 1 are considered as having a high Score). Its prognostic role has already been confirmed in aNSCLC patients treated with cytotoxic chemotherapy, epidermal growth factor receptor-tyrosine kinase inhibitors (EGFR-TKI) or second-line immunotherapy [17,20,21].

However, GRIm-Score has never been evaluated in a population of aNSCLC patients receiving first-line pembrolizumab monotherapy, and GRIm-Score early-on-treatment variation together with its potential prognostic/predictive value during pembrolizumab treatment is still to be investigated.

The aim of our study was to analyze the association of GRIm-Score at baseline (GRImT0), at 45 days since treatment initiation (GRImT1) and of its variation (GRImΔ = GRImT0 − GRImT1) with the clinical activity of pembrolizumab in a real-life setting. In order to assess GRIm-Score as a potential predictive biomarker, an additional cohort of chemotherapy-treated aNSCLC patients was also analyzed together with those receiving first-line pembrolizumab.

## 2. Materials and Methods

### 2.1. Ethics Statement

The study was conducted in accordance with the Declaration of Helsinki (as revised in 2013). Ethical approval to conduct this study was granted by the CERM (Comitato Etico Regione Marche) Ancona (Register number 984) and by the Medical Ethics Committee Erasmus MC (IDIGE study-Register number MEC-2020-0072) under a broader protocol to investigate clinical characteristics and gender differences in advanced/metastatic aNSCLC patients receiving first-line treatment. Informed consent was not required because of the retrospective nature of the study.

### 2.2. Study Population and Data Collection

Data from 135 consecutive aNSCLC patients with PD-L1 expression ≥ 50% and no EGFR mutation or ALK translocation, treated with first-line pembrolizumab monotherapy, from July 2017 to July 2020, at five European institutions, were retrospectively collected.

To determine whether the predictive value of the GRIm-Score is immunotherapy specific, a control cohort of 87 EGFR/ALK wild-type aNSCLC patients, treated with first-line platinum-based doublet chemotherapy, between February 2011 and May 2017, at Università Politecnica delle Marche was also collected. None of these patients had received ICIs as second or further lines treatment after progression to chemotherapy.

Demographic, clinical and pathological patient characteristics as well as blood count parameters, radiological response and survival data were retrieved from electronic medical records data. PD-L1 expression was assessed as routine care according to standard practice for each Institution. Response to both pembrolizumab and chemotherapy was evaluated according to institutional guidelines (no later than 3 months since treatment starting) as per RECIST criteria (version 1.1).

The GRIm-Score was calculated as previously described by Bigot et al. [20] based on NLR, LDH and serum albumin. Patients were assigned 1 point if they had NLR > 6, LDH > upper limit normal or albumin < 3.5 g/dL, for a total of 3 points. GRIm-Score < 2 was considered as a low score. The score was evaluated at baseline (GRImT0: namely within 2 weeks before the first infusion), 45 days since treatment initiation (GRImT1: namely at day 1 of cycle 3, before treatment administration) and the variation between the two timepoints was computed (GRImΔ: stable/positive GRImΔ meaning that GRIm-Score remained stable or decreased by at least one point while negative GRImΔ meaning that GRIm-Score increased between baseline and T1).

### 2.3. Study Objectives

The main objective of our study was to evaluate the correlation between GRImT0, GRImT1 and GRImΔ and clinical outcomes in a population of aNSCLC treated with first-line pembrolizumab. In order to further analyze the predictive role of GRImT0, GRImT1 and GRImΔ, their impact on aNSCLC patients treated with first-line chemotherapy was also assessed. The investigated clinical outcomes were objective response rate (ORR), median progression-free survival (PFS) and median overall survival (OS).

### 2.4. Statistical Analysis

Patient and disease characteristics were reported using descriptive statistics. Differences between groups were compared by the Pearson’s Chi-squared test or Fisher’s exact test for categorical variables and by two sample T-test or Wilcoxon rank-sum test for continuous instances. PD-L1 percentages were stratified into two categories and the cut-off value of 77.5% was obtained by receiver operating characteristic (ROC) curves considering disease response as event (Supplementary Figure S1).

ORR was defined as the proportion of patients who achieved tumor size reduction at the radiological assessment (either complete response (CR) or partial response (PR)). Association of variables with ORR was tested with univariate and multivariate logistic regression analyses.

Included patients were followed-up until death, loss of contact or data lock (July 2020). PFS and OS were calculated from the date of pembrolizumab/chemotherapy commencement until radiological progression or death/last follow up for PFS (censored at last follow-up for patients alive and without progression) and until death/last follow up for OS (censored at last follow-up for patients alive). PFS and OS were analyzed using the Kaplan–Meier method and differences in probability of surviving between the strata were evaluated by log-rank (Mantel–Cox) test.

Adjusted hazard ratios (HRs) together with 95% confidence intervals (CI) were calculated using a Cox multivariate proportional hazard regression model. The choice of the covariates to adjust for in multivariate analysis was based on their clinical relevance and statistical significance in a univariate analysis (*p* ≤ 0.1). No-multicollinearity of the covariates was checked.

To better compare the role of GRIm-Score in the two different cohorts of treated patients, a random case-control matching was performed, by randomly pairing all the cases (from the chemotherapy cohort) and controls (from the pembrolizumab cohort) on the basis of those characteristics which resulted significantly unbalanced between the two cohorts: GRImT0 (low vs. high), Eastern Cooperative Oncology Group (ECOG)–Performance Status (PS) (0 vs. ≥1) and disease burden (≤2 metastatic sites of disease vs. >2 sites). A caliper width of 0.2 for the standard deviation was used for the random case-control matching and patients who had missing data on the abovementioned characteristics were excluded. Statistical analyses were performed using R 3.6.0 (R Foundation for Statistical Computing, Free Software Foundation, Boston, MA, USA) and a significance two-tailed level of 0.05 was chosen to assess the statistical significance.

## 3. Results

### 3.1. Patient Characteristics

In total, 222 patients with aNSCLC from five different centers were retrospectively included in the study. The chemotherapy-treated patients were all collected from the same center. GRImT0, GRImT1 and GRImΔ were available for 175, 167 and 155 patients, respectively (Supplementary Figure S2). Sixty-one percent of included patients received pembrolizumab and 39% were treated with chemotherapy, as first-line therapy. Clinical and demographic patients’ characteristics by treatment cohort are summarized in Table 1. Median age was 70 years (range 36–91). One hundred and forty-three (64%) were males and seventy-nine (36%) were females. At baseline, PS ECOG was 0 and ≥ 1 in 32% and 65% of cases, respectively. Almost all patients considered for the analysis were smokers (smokers vs. non-smokers: 87% vs. 9%). In the overall population median follow up was 24.0 months (95% confidence interval (CI), 16.6–32.6). Median OS and PFS were 12.0 (95% CI, 5.5–27.5) and 6.5 months (95% CI, 2.9–13.3), respectively. In the pembrolizumab cohort, median OS was 15.6 and median PFS was 7.0 months. In the chemotherapy group, median OS and PFS were 9.2 and 6.4 months, respectively. In both cohorts, no correlation between GRImT0, GRImT1 and GRImΔ and baseline clinical pathological characteristics were noted, as shown in Supplementary Table S1.

### 3.2. Clinical Outcome According to GRImT0

**Figure 1:**
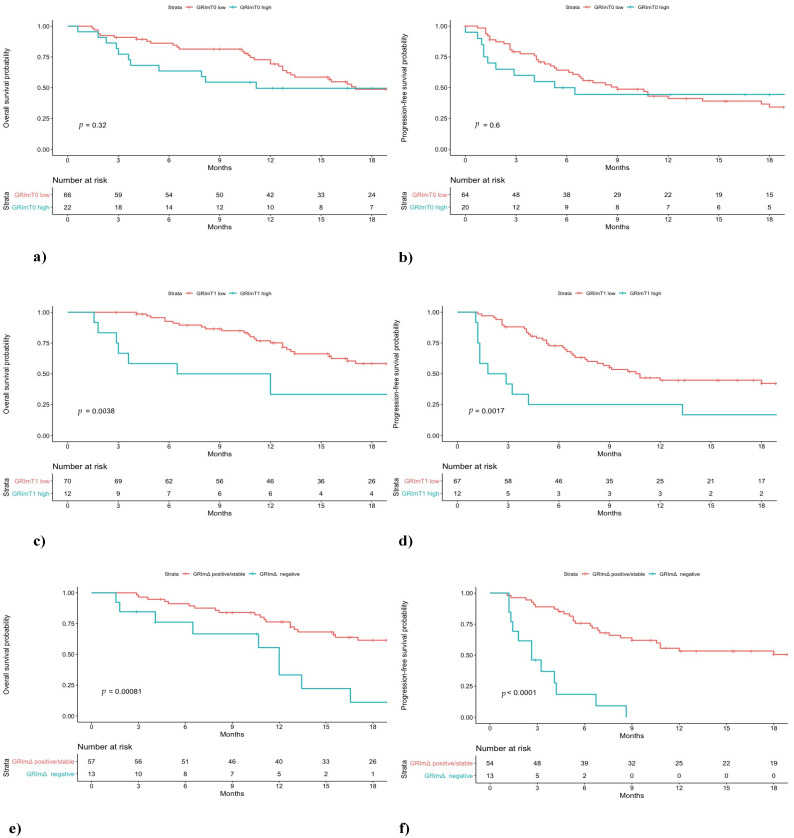
Overall survival (OS) and progression-free survival (PFS) of non-small cell lung cancer (NSCLC) patients treated with first-line pembrolizumab stratified by GRImT0 (**a**,**b**), GRImT1 (**c**,**d**) and GRImΔ (**e**,**f**) values.

**Figure 2:**
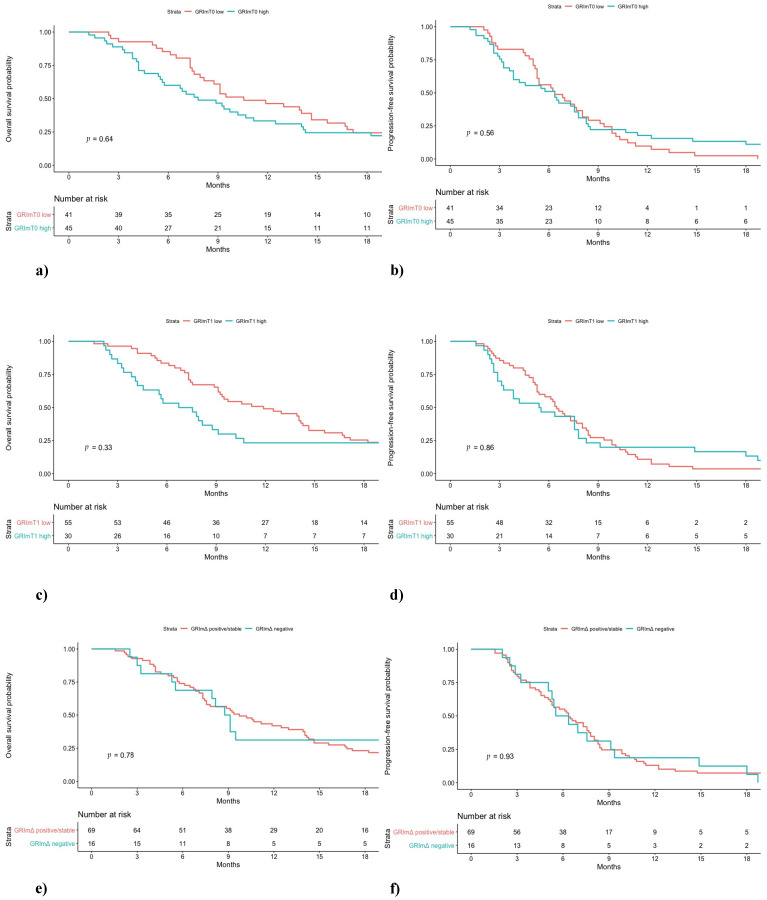
Overall survival (OS) and progression-free survival (PFS) of NSCLC patients treated with first-line chemotherapy stratified by GRImT0 (**a**,**b**), GRImT1 (**c**,**d**) and GRImΔ (**e**,**f**) values.

In the pembrolizumab cohort, a difference in low and high GRImT0 distribution was found, with 49% and 16% of patients, respectively. No significant difference was observed between low GRImT0 and high GRImT0 score both in terms of OS and PFS (low vs. high: median OS 17.0 vs. 11.2 months, *p* = 0.32; median PFS 9.0 vs. 5.9 months, *p* = 0.60; Figure 1). In the chemotherapy cohort, the proportion of patients with low and high GRImT0 was similar, 47% and 53%, respectively. Likewise, also in the chemotherapy group, no significant difference was found in terms of clinical outcome (low vs. high: median OS 10.6 vs. 7.8 months, *p* = 0.64; median PFS 6.4 vs. 6.4 months, *p* = 0.56; Figure 2). In addition, no significant variation was noticed in terms of ORR by GRImT0 in both cohorts (ORR 46.7% vs. 40%, *p* = 0.60; ORR 55.5% vs. 38.9%, *p* = 0.16).

### 3.3. Clinical Outcome According to GRImT1

Median OS and PFS of pembrolizumab-treated patients with low GRImT1 were significantly longer compared to patients from the same cohort with high GRImT1 (low vs. high: median OS not reached vs. 9.2 months, *p* < 0.01; median PFS 10.8 vs. 2.3 months, *p* < 0.01; Figure 1). Interestingly, in the chemotherapy-treated group, no significant difference both in OS and PFS was observed (low vs. high: median OS 11.9 vs. 7.1 months, *p* = 0.33; median PFS 6.5 vs. 5.5 months, *p* = 0.86; Figure 2). In terms of ORR at GRImT1, no variation in both cohorts was found (ORR 52.3% vs. 25%, *p* = 0.08; ORR 54.5% vs. 34.6%, *p* = 0.11).

### 3.4. Clinical Outcome According to GRImΔ

The outcome of patients receiving pembrolizumab with positive/stable GRImΔ was better compared to patients with negative GRImΔ in terms of OS and PFS (median OS not reached vs. 12.0 months, *p* < 0.01; median PFS 20.6 vs. 2.6 months, *p* < 0.01; Figure 1). Likewise, pembrolizumab patients with positive/stable GRImΔ showed a better ORR (ORR 58.2% vs. 7.6%, *p* < 0.01). Conversely, no difference in terms of OS, PFS (median OS 9.7 vs. 8.9 months, *p* = 0.78; median PFS 6.4 vs. 5.9 months, *p* = 0.93; Figure 2) and ORR (49% vs. 40%, *p* = 0.53) was demonstrated among chemotherapy-treated patients according to GRImΔ (positive/stable GRImΔ vs. negative GRImΔ). Details of univariate analysis in both cohorts are shown in Supplementary Table S2.

### 3.5. Multivariate Analysis and Case-Control Random Matching

The prognostic and predictive role of both GRImT1 and GRImΔ in the pembrolizumab-cohort was confirmed at multivariate analysis (high GRImT1 HR for death: 2.63, 95% CI 1.18–5.86, *p* = 0.01, negative GRImΔ HR for death: 3.28, 95% CI, 1.39–7.74, *p* < 0.01; high GRImT1 HR for progression: 3.05, 95% CI, 1.42–6.54, *p* < 0.01; negative GRImΔ HR for progression: 6.98, 95% CI, 2.94–16.61, *p* < 0.01). No significant association with ORR was found. Details of multivariate analysis are shown in Table 2 and Table 3.

After performing the case-control random matching, 55 patients from the pembrolizumab and chemotherapy cohorts respectively were paired, with no statistically significant differences between the characteristics of matched subjects. In the matched pembrolizumab cohort low GRImT1 retained its prognostic value in terms of OS and PFS but not ORR (median OS not reached vs. 3.6 months, *p* < 0.01; median PFS 18 months vs. 1.8, *p* < 0.01; ORR 54% vs. 22%, *p* = 0.09), and positive/stable GRImΔ resulted associated with OS, PFS and ORR (median OS not reached vs. 12.0 months, *p* = 0.01; median PFS 20.6 months vs. 1.8 months, *p* < 0.01; ORR 56% vs. 0%; *p* < 0.01) (Supplementary Figure S3). Again, no significant outcome differences were seen according to GRImT0 in this group.

On the contrary, in the matched chemotherapy cohort GRImT0, but not GRImT1 and GRImΔ, resulted prognostic. Particularly, patients with low GRImT0 showed better OS (median OS 11.9 months vs. 6.4, *p* = 0.01) and PFS (median PFS 6.8 months vs. 3.8, *p* < 0.01; Supplementary Figure S4).

## 4. Discussion

Prompt identification of aNSCLC patients who benefit from first-line pembrolizumab is crucial in clinical practice.

Our data show that GRImT1 and GRImΔ are more reliable biomarkers of outcome compared to GRImT0 in aNSCLC patients treated with pembrolizumab. Patients characterized by high GRImT1 and negative GRImΔ exhibited a worse clinical outcome compared to the ones who detained low GRImT1 and stable/positive GRImΔ.

On the other hand, in aNSCLC patients treated with first-line chemotherapy, no association between GRImT1, GRImΔ and patient outcome was found. Therefore, GRImT1 and GRImΔ acquired a prognostic and predictive role in aNSCLC patients receiving pembrolizumab, beyond PS, smoking status, disease burden and presence of liver metastases.

Immunotherapy revamped the course of patients affected by aNSCLC, becoming the treatment of choice as first-line monotherapy in case of PD-L1 expression ≥ 50% and in association with chemotherapy regardless of PD-L1. It is also an effective option for further lines beyond PD-L1 expression. However, a wide portion of patients do not benefit from immunotherapy and research is moving toward the detection of reliable tools for predicting treatment efficacy [8,21,22,23,24,25].

In this context, beside tumor tissue biomarkers such as PD-L1 staining, tumor mutational burden (TMB) [26] and immune cells infiltration [27], blood-derived parameters may represent a useful and easy-to-access predictor of immunotherapy response.

During the last years, several studies analyzed the relationship between blood parameters related to systemic inflammation (such as absolute immune cell count, NLR and LDH) and outcome of patients treated with immunotherapy by evaluating them at baseline [28,29], after several therapy cycles [30] and also assessing their variations during treatment [19,31].

Elevated LDH and NLR results are associated with poorer survival outcomes in patients treated with immunotherapy in phase I clinical trials, regardless of tumor type [32].

In our study we took into account NLR and LDH, in addition to blood albumin concentration, another well-established prognostic factor in cancer patients regardless of tumor type and oncological treatment [33], embedded together in the GRIm-Score.

Baseline GRIm-Score was firstly investigated in a cohort of 155 patients enrolled into ICIs treatment phase I trials [20]. Bigot et al. demonstrated the prognostic value of GRImT0 in this setting, suggesting its role as a tool for clinicians to better recognize patients who benefit from immunotherapy. Afterwards, the prognostic role of GRImT0 was demonstrated in a wide cohort of aNSCLC patients treated with first-line chemotherapy [34]. In our real-life study, GRImT0 did not result associated with the clinical outcome of aNSCLC patients treated with first-line pembrolizumab. In addition, after performing the case-control random matching, in our control cohort of chemotherapy-treated patients GRIm0 was correlated with clinical outcome, in line with the results of Minami et al. [34].

Prior to this study, no data were available regarding GRIm at day 45 or its variations during treatment cycles.

Recently, several works took into account other blood parameters score variations during ICIs treatment. Simonaggio et al. [19] showed in a cohort of aNSCLC and metastatic renal cell carcinoma (mRCC) receiving nivolumab that NLR variation represents an optimal and dynamic marker of treatment response. Likewise, Dusselier et al. [31] observed worse outcome in aNSCLC patients treated with nivolumab as second or further lines characterized by an increased NLR value between the 1st and 4th nivolumab infusion. In our study, GRImT1 and, above all, GRImΔ were able to predict patient outcome. Patients treated with pembrolizumab who showed negative GRImΔ detained worse PFS (median PFS 2.6 months vs. 10.8 months, *p* < 0.001) as well as reduced OS (median OS 12.0 months vs. not reached, *p* < 0.001) compared to those with positive/stable GRImΔ. In addition, patients with stable/positive GRImΔ between the two time points showed a better ORR (ORR 58.2% vs. 7.6%, *p* = 0.003): specifically, only 1 of the 13 patients who had a negative GRImΔ subsequently showed an OR. To note, the variation in GRIm score from 0 to 1 point of this patient was due to a “slight” increase in LDH (LDH at baseline: 227 UI/L vs. LDH at 45 days: 301 UI/L). These data suggest that the time-evolution of easy-to-use combination scores may better predict patient outcome under first-line pembrolizumab treatment.

Regarding validation chemotherapy patients, neither GRImT1 nor GRImΔ detained a prognostic value, thus reinforcing their predictive role. Noteworthy, the GRIm-Score was initially validated in a population of patients included in phase I trials [20], in which patients undergo radiological evaluation every 4–6 weeks. In the real-life setting, patients are revaluated less frequently. Therefore, recognizing which patients under immunotherapy are benefiting from treatment in the early treatment cycles becomes crucial.

By looking at the survival curves of pivotal clinical trials such as KEYNOTE-024 [8] and KEYNOTE-042 [22], rapid progression of disease in a subset of patients during the first months of treatment with immunotherapy is clearly evident and hyperprogressive disease has been reported in about 13–26% of aNSCLC patients [35]. Despite a high PD-L1 expression level, aNSCLC patients with PD-L1 ≥ 50% respond differently to pembrolizumab, and it becomes crucial to recognize those patients who might rather benefit from a first-line combination therapy. In our study we observed worse survival outcomes compared to the pivotal studies, yet consistent with other real-word experiences [4].

A recent work by Banna et al. suggested that low NLR < 5, when combined with high PD-L1 > 80% or normal LDH (nLDH < 269) represents an easily assessable tool to identify patients more likely to benefit from pembrolizumab monotherapy; conversely, patients with a high PD-L1 (> 80%), but NLR> 5 and/or high LDH may warrant a combination therapy [36]. In our study, GRImT1 and mostly GRImΔ, revealed a fundamental role in identifying patients with elevated PD-L1 staining who do not benefit from immunotherapy alone and might better respond to a combination treatment starting from the third cycle of therapy. Interestingly, in the analysis by Banna et al., the PD-L1 cut-off from the ROC curves was 77.5%, consistent to what we found in our population.

Our study has some limitations. First, it is a retrospective study with a restricted case series. Hence, our results need validation on larger and prospective trials. Second, GRIm parameters were not evaluable for some patients. Therefore, when just one of the parameters considered (NLR, albumin, LDH) was not available at T0 or T1, the GRImΔ was not calculated. Third, the two cohorts of patients considered were not homogeneous at baseline. Indeed, chemotherapy patients more frequently detained elevated GRImT0 (*p* < 0.01). A possible explanation could be the wider use of corticosteroids in patients treated with cytotoxic agents, such as platinum-based chemotherapy combined with pemetrexed (corticosteroids are used in clinical practice for pemetrexed infusion premedication as well as for adverse effects prevention and management) [37]. This aspect could have led to an altered NLR and therefore to an increase of GRIm score, both at baseline and at 45 days. On the other hand, patients treated with pembrolizumab detained worse baseline clinical characteristics, such as higher ECOG-PS value and more metastatic sites, compared to those who received chemotherapy. However, the prognostic/predictive role of GRImT1 or GRImΔ in the pembrolizumab cohort was confirmed after making patients’ characteristics well balanced in a case control random matching. To note, since PD-L1 status of chemotherapy-treated patients was not available, we did not correct for this variable in the chemotherapy cohort. We also conducted multivariable analysis to adjust for other significant prognostic factors in aNSCLC, which still confirmed our findings.

Altogether, our results showed that GRImT1 and GRImΔ might represent easy-to-access and reliable tools to identify which aNSCLC patients with PD-L1 > 50% are benefiting from pembrolizumab monotherapy. Remarkably, our work proved that most of the patients with negative GRImΔ during immunotherapy did not show an objective response at subsequent tumor imaging. Therefore, these data suggest that GRIm-Score improvement under pembrolizumab treatment could be helpful in driving clinical decisions in those cases of dubious progression at first radiological evaluation.

In real-world clinical practice, first radiological evaluation is rarely performed earlier than 3 months after the start of treatment, thus emphasizing the importance of an early prognostic biomarker, and patients with early-on-treatment negative GRImΔ should probably be promptly referred to a combination therapy.

## Figures and Tables

**Table 1:** Patient characteristics by treatment cohort.

Characteristic	Pembrolizumab No. (%)	Chemotherapy No. (%)	*p-*Value ^a^
Total	135 (61)	87 (39)	
Median age (range)	71 (44–91)	73 (36–88)	0.12
Sex			
Male	84 (62)	59 (68)	
Female	51 (38)	28 (32)	0.48
ECOG PS			
0–1	103 (76)	81 (93)	
≥2	26 (19)	6 (7)	0.01
Unknown	6 (5)	0 (0)	
Smoking status			
Never smoker	16 (12)	5 (6)	
Current/former smoker	116 (86)	77 (88)	0.22
Unknown	3 (2)	5 (6)	
Tumor histotype			
Adenocarcinoma	113 (84)	68 (78)	
Non Adenocarcinoma	18 (13)	09 (10)	0.83
Unknown	4 (3)	10 (12)	
PD-L1			
50–77%	56 (42)	NA	
78–100%	55 (40)	NA	NA
>50 % (not otherwise specified)	24 (18)	NA	
Number of metastatic sites			
≤2 metastatic sites	73 (54)	62 (71)	
>2 metastatic sites	62 (46)	25 (29)	0.01
Metastatic sites			
Brain metastases			
No	112 (83)	71 (82)	
Yes	23 (17)	16 (18)	0.93
Liver metastases			
No	113 (84)	75 (86)	
Yes	22 (16)	12 (14)	0.75
Bone metastases			
No	93 (69)	57 (65)	
Yes	42 (31)	30 (35)	0.70
GRImT0			
Low (0–1)	66 (49)	41 (47)	
High (2–3)	22 (16)	45 (53)	<0.01
Unknown	47 (35)	0 (0)	
GRImT1			
Low (0–1)	70 (52)	55 (63)	
High (2–3)	12 (8)	30 (35)	<0.01
Unknown	53 (40)	2 (2)	
GRImΔ			
Positive/stable Negative Unknown	57 (42) 13 (10) 65 (48)	69 (79) 16 (18) 2 (2)	1
ORR			
CR/PR	75 (55)	34 (40)	
SD/PD	45 (33)	38 (44)	<0.01
Unknown	15 (12)	15 (16)	
Median PFS (months)	7.0 (95% CI 5.3–12.0)	6.4 (95% CI 5.3–7.6)	<0.01
Median OS (months)	15.6 (95% CI 12.0–NR)	9.2 (95% CI 7.6–13.0)	<0.01

No., Number; ECOG PS, Eastern Cooperative Oncology Group performance status; PD-L1, programmed death-ligand 1; GRImT0, GRIm-Score at baseline; GRImT1, GRIm-Score 45 days since treatment initiation; GRImΔ, GRIm-Score variation between the two timepoints; ORR, objective response rate; CR, complete response; PR, partial response; SD, stable disease; PD, progressive disease; PFS, progression-free survival; CI, confidence interval; OS, overall survival; NR, not reached. ^a^ Chi-squared test comparing proportions between pembrolizumab and chemotherapy groups. *p*-values were calculated excluding unknown values and considered statistically significant if *p* < 0.05.

**Table 2:** Multivariable analyses for ORR, PFS and OS in pembrolizumab cohort considering GRImT1 as independent variable.

Test Variables	ORR OR (95% CI)	*p*-Value	PFS HR (95% CI)	*p*-Value	OS HR (95% CI)	*p*-Value
GRImT1 low (ref.)/high	0.79 (0.52–1.20)	0.29	3.05 (1.42–6.54)	<0.01 ^a^	2.63 (1.18–5.86)	0.01 ^a^
Never smokers (ref.)/ Current-former smokers	1.19 (0.71–1.96)	0.50	0.97 (0.29–3.26)	0.96	0.41 (0.12–1.44)	0.16
ECOG PS 0–1 (ref.)/≥2	0.83 (0.57–1.20)	0.34	1.29 (0.61–2.72)	0.49	1.14 (0.46–2.81)	0.76
Non adenocarcinoma (ref.)/ Adenocarcinoma	0.71 (0.49–1.02)	0.07	–	–	–	–
Metastatic sites ≤2/>2	–	–	2.18 (1.14–4.17)	0.02 ^a^	1.75 (0.88–3.49)	0.10
PD-L1 percentage <78/≥78	1.32 (1.02–1.71)	0.04 ^a^	–	–	–	–
Liver metastases No. (ref.)/Yes	–	–	1.24 (0.52–2.96)	0.61	–	–
Bone metastases No. (ref.)/Yes	0.79 (0.59–1.06)	0.12	–	–	–	–

CI, confidence interval; ORR, objective response rate; OR, odds ratio; PFS, progression-free survival; HR, hazard ratio; OS, overall survival; GRImT1, GRIm-Score 45 days since treatment initiation; ECOG PS, Eastern Cooperative Oncology Group performance status; PD-L1, programmed death-ligand 1; No., number. ^a^ Statistically significant (*p* < 0.05).

**Table 3:** Multivariable analyses for ORR, PFS and OS in pembrolizumab cohort considering GRImΔ as independent variable.

Test Variables	ORR OR (95% CI)	*p*-Value	PFS HR (95% CI)	*p*-Value	OS HR (95% CI)	*p*-Value
GRImΔ positive-stable (ref.)/negative	0.74 (0.50–1.08)	0.12	6.98 (2.94–16.61)	<0.01 ^a^	3.28 (1.39–7.74)	<0.01 ^a^
Never smokers (ref.)/ Current-former smokers	1.13 (0.67–1.89)	0.64	0.83 (0.24–2.90)	0.78	0.34 (0.09–1.21)	0.10
ECOG PS 0–1 (ref.)/≥2	0.87 (0.58–1.29)	0.50	0.95 (0.38–2.38)	0.92	0.81 (0.27–2.43)	0.71
Non adenocarcinoma (ref.)/ Adenocarcinoma	0.79 (0.54–1.15)	0.22	–	–	–	–
Metastatic sites ≤2/>2	–	–	1.28 (0.59–2.77)	0.52	1.15 (0.54–2.47)	0.70
PD-L1 percentage <78/≥78	1.21 (0.91–1.60)	0.17	–	–	–	–
Liver metastases No. (ref.)/Yes	–	–	2.14 (0.85–5.40)	0.10	–	–
Bone metastases No. (ref.)/Yes	0.85 (0.62–1.15)	0.30	–	–	–	–

CI, confidence interval; ORR, objective response rate; OR, odds ratio; PFS, progression-free survival; HR, hazard ratio; OS, overall survival; GRImΔ, GRIm-Score variation between the two timepoints; ECOG PS, Eastern Cooperative Oncology Group performance status; PD-L1, programmed death-ligand 1; No., number. ^a^ Statistically significant (*p* < 0.05).

## Data Availability

The data that support the findings of this study are available from the corresponding author, RB, upon reasonable request.

## Supplementary Materials

The following are available online at https://www.mdpi.com/2077-0383/10/5/1005/s1, Figure S1: Receiver operating characteristic (ROC) curve of PD-L1 on disease response (patients treated with pembrolizumab), Figure S2: Flow diagram of study population, Figure S3: Overall survival (OS) and progression-free survival (PFS) of NSCLC patients treated with first-line pembrolizumab stratified by GRImT0 (a,b), GRImT1 (c,d) and GRImΔ (e,f) values, after performing the case-control random matching, Figure S4: Overall survival (OS) and progression-free survival (PFS) of NSCLC patients treated with first-line chemotherapy stratified by GRImT0 (a,b), GRImT1 (c,d) and GRImΔ (e,f) values, after performing the case-control random matching, Table S1: Association between GRImT0, GRImT1, GRImΔ and key clinicopathologic features of pembrolizumab and chemotherapy patients, Table S2: Univariate analyses for ORR, PFS and OS in pembrolizumab and chemotherapy cohort.
