# Supplementary material for: The Gustave Roussy Immune (GRIm)-Score Variation Is an Early-on-Treatment Biomarker of Outcome in Advanced Non-Small Cell Lung Cancer (NSCLC) Patients Treated with First-Line Pembrolizumab
Source: J Clin Med. 2021 Mar 2;10(5):1005. doi: 10.3390/jcm10051005 (PMC7958321; doi:10.3390/jcm10051005)
Supplement: Supplementary file 1 [file jcm-10-01005-s001.zip › OTHER5-4199.docx]

**Supplementary Table S1. Association between GRIm T0, GRIm T1, GRImΔ and key clinicopathologic features of pembrolizumab and chemotherapy patients.**

|  | | **GRIm T0** | | **p value^a^** | **GRIm T1** | | **p value^a^** | **GRImΔ** | | **p value^a^** |
| --- | --- | --- | --- | --- | --- | --- | --- | --- | --- | --- |
|  |  | **Low (%)** | **High (%)** |  | **Low (%)** | **High (%)** |  | **Positive/stable (%)** | **Negative (%)** |  |
| **Pembrolizumab group** | | | | | | | | | | |
| **Age** | <65  ≥65 | 18 (27)  48 (73) | 5 (23)  17 (73) | 0.33 | 22 (27)  48 (73) | 3 (25)  9 (75) | 0.06 | 16 (28)  41 (72) | 3 (23)  10 (73) | 0.12 |
| **Sex** | Male  Female | 38 (58)  28 (42) | 17 (77)  5 (23) | 0.16 | 44 (63)  26 (37) | 10 (83)  2 (17) | 0.29 | 34 (60)  23 (40) | 10 (77)  3 (23) | 0.40 |
| **Smoke** | Current/Former smoker  Never smoker | 57 (90)  6 (10) | 20 (91)  2 (9) | 1 | 65 (96)  3 (4) | 9 (82)  2 (18) | 0.53 | 53 (96)  2 (4) | 10 (71)  4 (29) | 0.61 |
| **ECOG-PS** | 0-1  ≥2 | 52 (82)  11 (17) | 16 (76)  5 (24) | 0.75 | 58 (84)  11 (16) | 10 (83)  2 (17) | 1 | 48 (87)  7 (13) | 10 (77)  3 (23) | 0.61 |
| **Hystotype** | Adenocarcinoma  Non adenocarcinoma | 56 (86)  9 (14) | 14 (70)  6 (30) | 0.19 | 59 (85)  10 (15) | 8 (73)  3 (27) | 0.53 | 45 (80)  11 (20) | 12 (100)  0 (0) | 0.21 |
| **Number of metastatic sites** | <2  ≥2 | 32 (48)  34 (52) | 9 (41)  13 (59) | 0.71 | 38 (54)  32 (46) | 6 (50)  6 (50) | 1 | 32 (56)  25 (44) | 4 (31)  9 (69) | 0.18 |
| **Brain metastases** | No  Yes | 53 (75)  18 (25) | 13 (76)  4 (24) | 1 | 55 (79)  15 (21) | 11 (92)  1 (8) | 0.50 | 45 (74)  12 (26) | 12 (93)  1 (7) | 0.47 |
| **Liver metastases** | No  Yes | 58 (88)  8 (12) | 15 (65)  7 (35) | 0.07 | 59 (84)  11 (16) | 12 (100)  0 (0) | 0.31 | 47 (82)  10 (18) | 12 (93)  1 (7) | 0.65 |
| **Bone metastases** | No  Yes | 48 (73)  18 (27) | 11 (50)  11 (50) | 0.08 | 52 (74)  18 (26) | 8 (67)  4 (33) | 0.84 | 43 (75)  14 (25) | 7 (54)  6 (46) | 0.22 |
| **Chemotherapy group** | | | | | | | | | | |
| **Age** | <65  ≥65 | 8 (20)  31 (80) | 12 (26)  34 (74) | 0.9 | 13 (25)  39 (75) | 3 (10  27 (90) | 0.25 | 17 (25)  52 (75) | 0 (0)  14 (100) | 0.90 |
| **Sex** | Male  Female | 24 (58)  17 (42) | 35 (76)  11 (24) | 0.13 | 34 (62)  21 (38) | 23 (74)  7 (26) | 0.25 | 46 (67)  23 (33) | 11 (69)  5 (31) | 1 |
| **Smoke** | Current/Former smoker  Never smoker | 37 (95)  2 (5) | 40 (93)  3 (7) | 1 | 47 (90)  5 (10) | 29 (100)  0 (0) | 0.21 | 60 (92)  5 (8) | 16 (100)  0 (0) | 0.57 |
| **ECOG-PS** | 0-1  ≥2 | 39 (95)  2 (5) | 42 (91)  4 (9) | 0.78 | 54 (95)  3 (5) | 28 (93)  2 (7) | 1 | 65 (94)  4 (6) | 15 (94)  1 (6) | 1 |
| **Hystotype** | Adenocarcinoma  Non adenocarcinoma | 34 (90)  4 (10) | 34 (87)  5 (13) | 1 | 41 (85)  7 (15) | 26 (93)  2 (7) | 0.55 | 52 (87)  8 (13) | 15 (94)  1 (6) | 0.73 |
| **Number of metastatic sites** | <2  ≥2 | 30 (73)  11 (27) | 32 (70)  14 (30) | 0.89 | 43 (78)  12 (22) | 18 (60)  12 (40) | 0.13 | 52 (75)  17 (25) | 9 (56)  7 (44) | 0.22 |
| **Brain metastases** | No  Yes | 36 (88)  5 (12) | 35 (76)  11 (24) | 0.26 | 44 (80)  11 (20) | 26 (87)  4 823) | 0.64 | 55 (80)  14 (20) | 15 (94)  1 (6) | 0.33 |
| **Liver metastases** | No  Yes | 36 (88)  5 (12) | 39 (85)  7 (15) | 0.93 | 49 (66)  25 (34) | 6 (55)  5 (45) | 0.68 | 61 (88)  8 (12) | 13 (81)  3 (19) | 0.72 |
| **Bone metastases** | No  Yes | 26 (63)  15 (37) | 31 (67)   \| 15 (33) \| \| --- \| | 0.87 | 39 (71)  16 (29) | 16 (53)  14 (47) | 0.17 | 46 (67)  23 (23) | 9 (56)  7 (44) | 0.62 |

Abbreviations: GRImT0, GRIm-score at baseline; GRImT1, GRIm-score 45 days since treatment initiation; GRImΔ, GRIm-score variation between the two timepoints; ECOG PS, Eastern Cooperative Oncology Group performance status.

^a^ P values were calculated excluding unknown values and considered statistically significant if p < 0.05.

**Supplementary Table S2. Univariate analyses for ORR, PFS and OS in pembrolizumab and chemotherapy cohort.**

|  | **Univariate analysis** | | | | | |
| --- | --- | --- | --- | --- | --- | --- |
| **Test variables** | **ORR OR (95% CI)** | **p value** | **PFS HR (95% CI)** | **p value** | **OS HR (95% CI)** | **p value** |
| **Pembrolizumab cohort** | | | | | | |
| GRImT0 low (ref.)/high | 0.93 (0.72-1.20) | 0.60 | 1.19 (0.62-2.28) | 0.60 | 1.40 (0.71-2.73) | 0.32 |
| GRImT1 low (ref.)/high | 0.76 (0.56-1.03) | 0.08 | 2.93 (1.45-5.94) | <0.01^a^ | 2.93 (1.37-6.27) | <0.01^a^ |
| GRImΔ positive-stable (ref.)/negative | 0.56 (0.39-0.82) | <0.01^a^ | 7.36 (3.42-15.85) | <0.01^a^ | 3.61 (1.62-8.03) | <0.01^a^ |
| Age <65 (ref.)/≥65 | 1.04 (0.86-1.26) | 0.66 | 0.95 (0.57-1.57) | 0.84 | 1.24 (0.70-2.19) | 0.44 |
| Female (ref.)/Male | 0.97 (0.81-1.16) | 0.81 | 1.07 (0.67-1.70) | 0.77 | 1.24 (0.75-2.07) | 0.39 |
| Never smokers (ref.)/Current-Former smokers | 1.25 (0.96-1.61) | 0.09 | 0.54 (0.27-1.06) | 0.08 | 0.45 (0.22-0.89) | 0.02^a^ |
| ECOG PS 0-1 (ref.)/≥2 | 0.78 (0.63-0.97) | 0.03^a^ | 1.88 (1.10-3.21) | 0.02^a^ | 2.17 (1.26-3.72) | <0.01^a^ |
| Non adenocarcinoma (ref.)/Adenocarcinoma | 0.76 (0.59-0.99) | 0.04^a^ | 0.98 (0.51-1.87) | 0.96 | 0.76 (0.39-1.46) | 0.41 |
| Metastatic sites ≤2/>2 | 0.87 (0.73-1.04) | 0.13 | 2.39 (1.49-3.81) | <0.01^a^ | 1.72 (1.06-2.80) | 0.03^a^ |
| PD-L1 percentage <78/≥78 | 1.23 (1.01-1.49) | 0.03^a^ | 0.85 (0.50-1.42) | 0.54 | 0.71 (0.41-1.21) | 0.21 |
| Brain metastases No (ref.)/Yes | 1.04 (0.83-1.30) | 0.71 | 0.73 (0.39-1.36) | 0.32 | 0.61 (0.29-1.29) | 0.20 |
| Liver metastases No (ref.)/Yes | 0.97 (0.76-1.22) | 0.80 | 1.75 (1.01-3.05) | 0.04^a^ | 1.51 (0.84-2.74) | 0.16 |
| Bone metastases No (ref.)/Yes | 0.79 (0.65-0.96) | 0.02^a^ | 1.35 (0.83-2.22) | 0.22 | 1.31 (0.80-2.16) | 0.27 |
| **Chemotherapy cohort** | | | | | | |
| GRImT0 low (ref.)/high | 0.84 (0.67-1.06) | 0.16 | 0.87 (0.56-1.36) | 0.56 | 1.09 (0.71-1.69) | 0.64 |
| GRImT1 low (ref.)/high | 0.81 (0.64-1.04) | 0.11 | 0.96 (0.60-1.52) | 0.86 | 1.25 (0.79-1.97) | 0.33 |
| GRImΔ positive-stable (ref.)/negative | 0.91 (0.68-1.21) | 0.53 | 1.02 (0.58-1.77) | 0.93 | 0.92 (0.53-1.60) | 0.78 |
| Age <65 (ref.)/≥65 | 0.96 (0.72-1.27) | 0.80 | 0.86 (0.51-1.45) | 0.57 | 0.89 (0.52-1.50) | 0.66 |
| Female (ref.)/Male | 0.89 (0.69-1.16) | 0.41 | 1.06 (0.67-1.67) | 0.79 | 1.22 (0.77-1.94) | 0.39 |
| Never smokers (ref.)/Current-Former smokers | 0.87 (0.55-1.38) | 0.57 | 0.73 (0.29-1.81) | 0.50 | 0.72 (0.29-1.82) | 0.49 |
| ECOG PS 0-1 (ref.)/≥2 | 0.71 (0.47-1.08) | 0.12 | 2.69 (1.15-6.26) | 0.02^a^ | 3.16 (1.34-7.44) | <0.01^a^ |
| Non adenocarcinoma (ref.)/Adenocarcinoma | 1.04 (0.70-1.55) | 0.82 | 0.53 (0.25-1.09) | 0.09 | 0.60 (0.29-1.23) | 0.16 |
| Metastatic sites ≤2/>2 | 0.82 (0.63-1.05) | 0.13 | 1.68 (1.02-2.75) | 0.04^a^ | 1.75 (1.08-2.85) | 0.02^a^ |
| PD-L1 percentage <78/≥78 | NA | NA | NA | NA | NA | NA |
| Brain metastases No (ref.)/Yes | 1.03 (0.75-1.41) | 0.83 | 0.61 (0.33-1.12) | 0.11 | 0.88 (0.50-1.55) | 0.66 |
| Liver metastases No (ref.)/Yes | 0.85 (0.60-1.21) | 0.38 | 1.51 (0.80-2.84) | 0.19 | 1.49 (0.80-2.78) | 0.20 |
| Bone metastases No (ref.)/Yes | 1.14 (0.89-1.45) | 0.28 | 0.92 (0.59-1.45) | 0.74 | 1.46 (0.92-2.31) | 0.10 |

Abbreviations: CI, confidence interval; ORR, objective response rate; OR, odds ratio; PFS, progression-free survival, HR, hazard ratio; OS, overall survival; GRImT0, GRIm-score at baseline; GRImT1, GRIm-score 45 days since treatment initiation; GRImΔ, GRIm-score variation between the two timepoints; ECOG PS, Eastern Cooperative Oncology Group performance status; PD-L1, programmed death-ligand 1.

^a^ Statistically significant (p <0.05).
