# Supplementary figures and images for: The Gustave Roussy Immune (GRIm)-Score Variation Is an Early-on-Treatment Biomarker of Outcome in Advanced Non-Small Cell Lung Cancer (NSCLC) Patients Treated with First-Line Pembrolizumab
Source: J Clin Med. 2021 Mar 2;10(5):1005. doi: 10.3390/jcm10051005 (PMC7958321; doi:10.3390/jcm10051005)

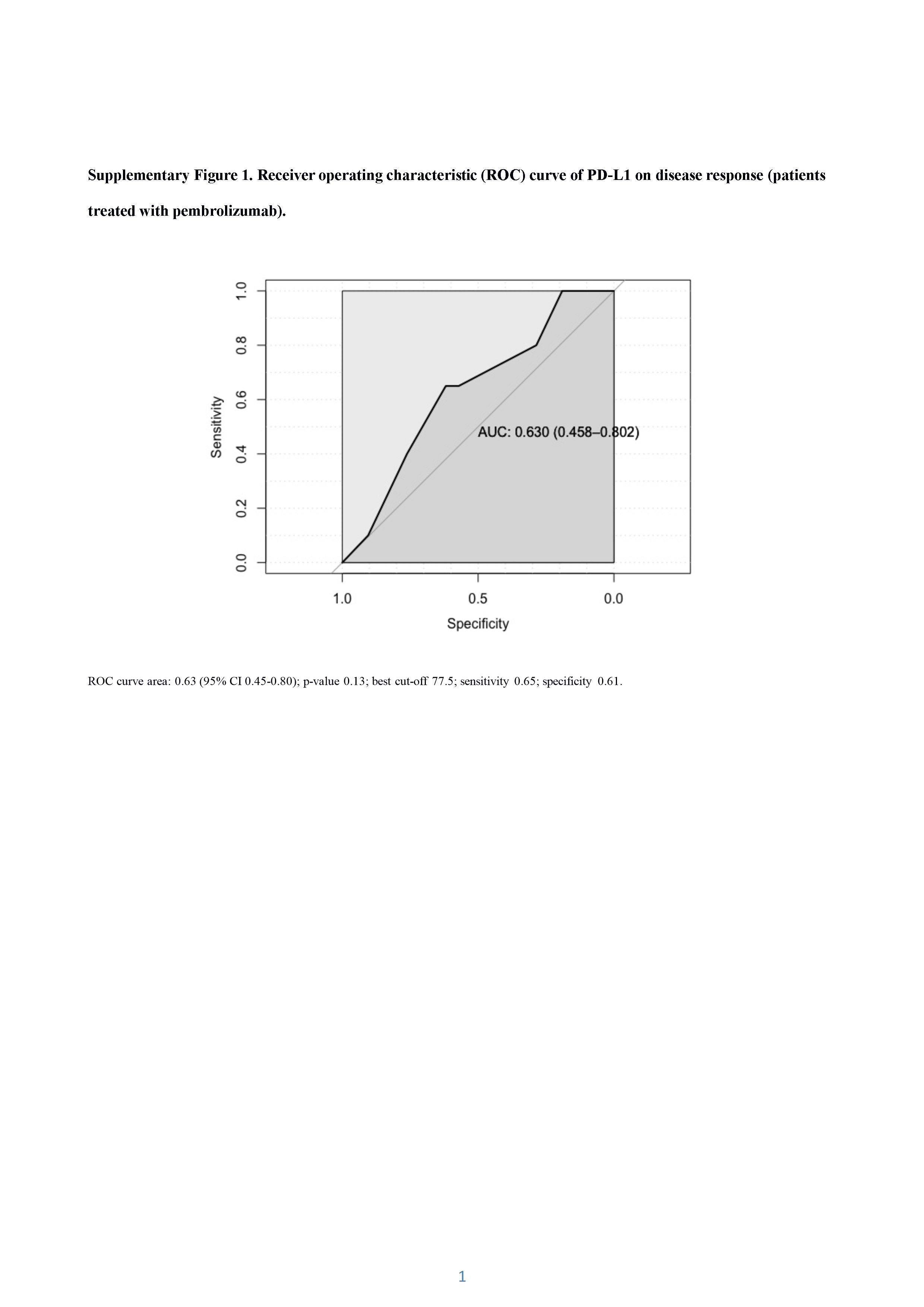

Supplement: Supplementary file 1 [file jcm-10-01005-s001.zip › -OTHER1-1241.jpg]

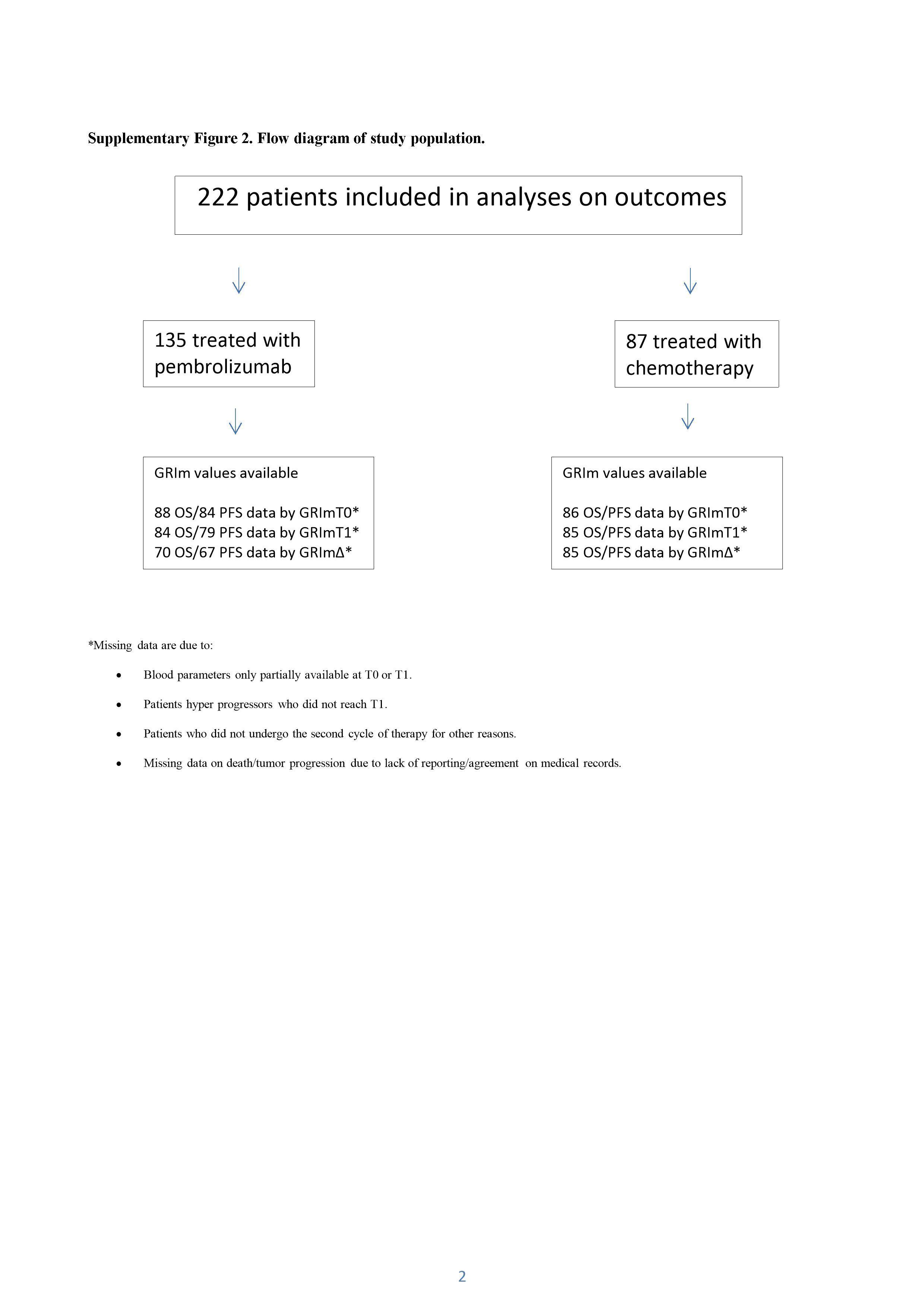

Supplement: Supplementary file 1 [file jcm-10-01005-s001.zip › OTHER2-3593.jpg]

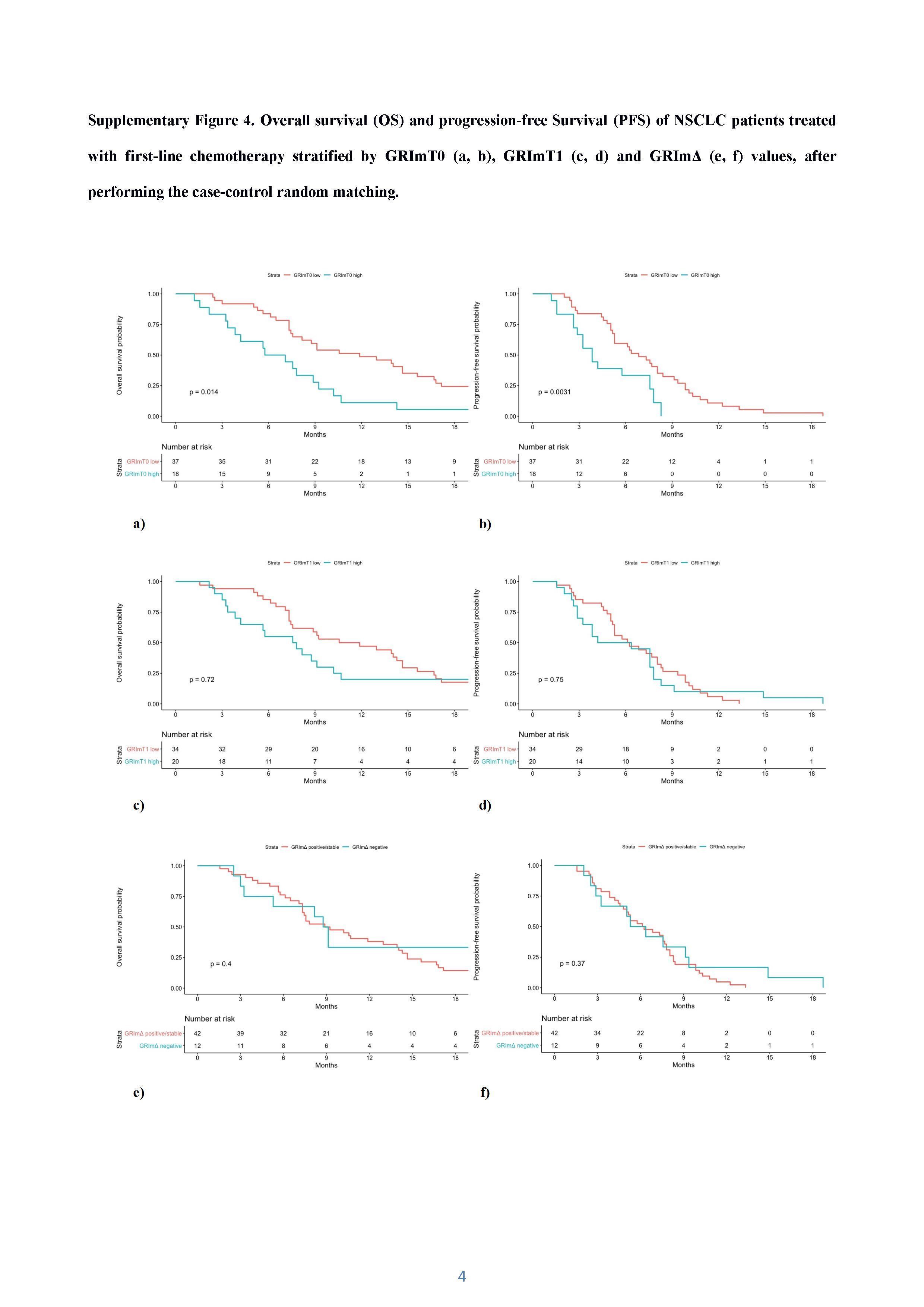

Supplement: Supplementary file 1 [file jcm-10-01005-s001.zip › OTHER3-3530.jpg]

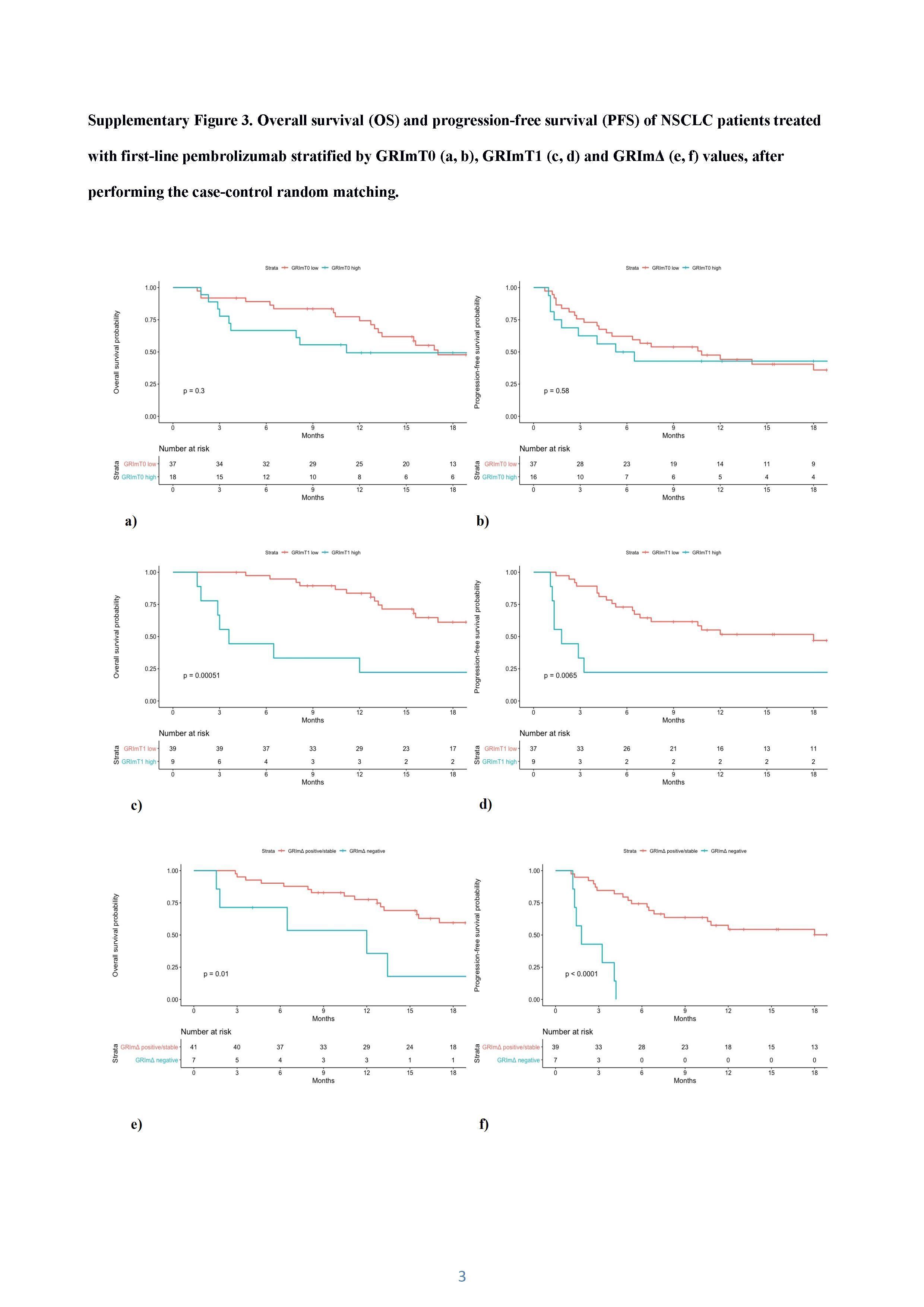

Supplement: Supplementary file 1 [file jcm-10-01005-s001.zip › OTHER4-3816.jpg]
